# Flexible integration of natural stimuli by auditory cortical neurons

**DOI:** 10.1152/jn.00200.2025

**Published:** 2026-04-21

**Authors:** Grace Wan Yu Ang, Claudia Clopath, Andriy S. Kozlov

**Affiliations:** 1 Department of Bioengineering, Imperial College London

## Abstract

Neurons have rich input-output functions for processing and combining their inputs. Although many experiments characterize these functions by directly activating synaptic inputs on dendrites *in vitro*, the integration of spatiotemporal inputs representing real-world stimuli is less well studied. Using ethologically relevant stimuli, we study neuronal integration in relation to Boolean AND and OR operations thought to be important for pattern recognition. We recorded single-unit responses in the mouse auditory cortex to pairs of ultrasonic mouse vocalization (USV) syllables. We observed a range of integration responses, spanning the sublinear to supralinear regimes, with many responses resembling the MAX-like function, an instantiation of the OR operation. Integration was more MAX-like for strongly activating features, and more AND-like for spectrally distinct inputs. Importantly, single neurons could implement more than one integration function, in contrast to artificial networks which typically fix activation functions across all units and inputs. To understand the mechanism underlying the flexibility and heterogeneity in neuronal integration, we modelled how dendritic properties could influence the integration of inputs with complex spectrotemporal structure. Our results link nonlinear integration in dendrites to single-neuron computations for pattern recognition.

## 1 Introduction

Network computations are shaped by how single neurons integrate their inputs to produce output signals. The input-output functions of neurons have been shown to be highly diverse [1–3] and dynamic [4], enabling the network to perform rich and complex tasks [5, 6]. To investigate neuronal integration, numerous studies have stimulated dendrites with well-controlled patterns of synaptic inputs *in vitro* [1, 3, 7–9] or measured responses to simple stimuli [10, 11] such as tone pips [12]. While these approaches have provided essential insights into the rules and biophysical mechanisms governing the integration of inputs, there is a gap in understanding how neurons combine natural, complex stimuli *in vivo*.

Single-neuron transfer functions can be approximated as logical operations used in computations [13, 14]. Among these, the OR and AND logical operations are thought to be particularly relevant for pattern recognition in sensory processing. The AND operation increases feature complexity for selectivity by summing over inputs encoding simpler features. On the other hand, MAX-pooling, an instantiation of the logical OR operation, confers invariance as the neuron’s response is primarily determined by its strongest input. These feature recombination functions were first demonstrated in the cat visual cortex [15] and more recently in the central auditory system of songbirds [16], with the latter study employing ethologically relevant stimuli to probe pooling responses. In the auditory system, such feature recombination functions may play an important role in auditory scene analysis. The AND operation, by constructing more refined representations that increase selectivity, would facilitate the discrimination of one auditory stimulus from another (e.g., distinguishing "cat" from "hat"). The OR operation, by supporting invariance, helps maintain recognition of a stimulus despite non-meaningful variation, and may help in filtering out irrelevant sensory input in complex auditory scenes.

In this study, our goal is to bridge the two complementary perspectives on neuronal input integration: the bottom-up approach focusing on the biophysical mechanisms that transform synaptic inputs to outputs, and the top-down perspective on computations performed by neurons to achieve selectivity and invariance. By recording extracellular single unit spikes in mouse auditory cortex, we measure integration responses to pairs of ultrasonic mouse vocalization (USV) syllables. We then model the integration of these ethologically relevant stimuli using a simple neuron model. This allows us to explore how the spatiotemporal pattern of synaptic inputs arriving onto dendrites shape the neuron’s resultant feature integration operation.

## 2 Materials and Methods

### 2.1 Surgical preparation

All procedures were carried out under the terms and conditions of licences issued by the UK Home Office under the Animals (Scientific Procedures) Act 1986. Extracellular recordings were made in the auditory cortex of adult female C57BL/6 mice (N=21, aged 6 to 11 weeks). Animals were anaesthetized using a mixture of fentanyl, midazolam and medetomidine (0.05, 5 and 0.5 mg/kg, respectively). A small craniotomy was made over the auditory cortex, at the caudal end of the left squamosal suture, centered 1.5 mm rostral to the lamdoid suture. The dura was removed and the surface of the brain was coated in a layer of silicon oil. The head was fixed to a rigid clamp and the mouse was transferred to an acoustic chamber.

### 2.2 Electrophysiological recording

Extracellular signals were recorded using a single-shank 32-channel silicon multi-electrode probe (Neuronexus), and acquired at a sample rate of 30 kHz (Neuronexus Smartbox Pro). The shank was lowered slowly and allowed to settle for at least 30 min before each recording. Spikes were extracted using the automatic spike sorting algorithm Kilosort3 (https://github.com/MouseLand/Kilosort) and manually curated with Phy (https://github.com/cortex-lab/phy). Units were considered well-isolated single units if they showed clear refractory periods in their autocorrelogram and had fewer than 1% spikes within a 1-ms interspike interval. Only stable units that exhibited little mechanical drift and that showed robust responses to repetitions of mouse USVs were identified for subsequent stages of the experiment.

### 2.3 Stimuli

Stimuli were delivered through an Avisoft UltraSoundGate Player (Avisoft Bioacoustics) connected to a PC. The sound level was adjusted such that the peak intensty did not exceed 85 dB SPL. To identify syllables for the summation index (SmI) measurements, a USV stimulus consisting of concatenated mouse vocalization song bouts [17] was presented for 10-15 repetitions. Syllables, approximately 100 ms in duration (based on the average duration of individual sylables) eliciting peaks in the PSTH were clipped and a 5-ms cosine ramp was applied to the start and end of each clip. When superimposing syllables to measure the combined stimulus response, syllables were combined with different temporal alignments, shifting from - 20 ms to + 20 ms in 10 ms increments. This was to ensure that the MAX response was not an artifact from stimuli that were misaligned in time, which would produce a smaller than expected response. All stimuli were presented for 10-15 repetitions and in a randomized order, with a 1-s interval between each presentation. Routines to create and manipulate the stimuli were written in Python; pitch-shifting was performed using the Librosa library.

### 2.4 Protocol for cross-adaptation

For cross-adaptation stimuli, the deviant syllable was concatenated with the train of adapting syllables with a 15-ms silence between each syllable. The order of the two syllables was then reversed to test adaptation in the following sequence. We measured the degree of cross-stimulus adaptation by calculating the ratio of the response to the deviant syllable before and after adaptation to the other syllable, where a lower ratio indicates greater adaptation. We defined asymmetric input independence as a ratio of at least 0.6 in one sequence, and bidirectional input independence as a ratio of at least 0.6 in both sequences.

### 2.5 Analyses

#### Summation Index (SmI)

The summation index described in previous studies was used as a metric to quantify feature recombination functions [15, 16, 18]:

(1) $$SmI=\frac{R_{a+b}-MAX(R_{a},R_{b})}{MIN(R_{a},R_{b})}$$

where *R*_*a*+*b*_ is the response to the combined stimulus (superimposed syllables) and *R*_*a*_ and *R*_*b*_ the responses to individual stimuli. Table 1 shows the range of SmIs corresponding to each integration function.

Stimuli pairs for SmI calculations were included in the analyses if the response to each syllable was significantly greater than spontaneous activity (500 ms before stimulus onset). When individual responses did not meet this criterion, the stimulus pair was included if the firing rate to the combined stimulus was at least 25% higher than the dominant response to individual stimuli.

#### Pitch selectivity

When responses to *n* pitch-shifted versions of a syllable were recorded, pitch selectivity, *S*, was calculated using a metric previously described in [19]:

(2) $$S=\frac{1-{\left(\sum \frac{r_{i}}{n}\right)}^{2}/\sum (\frac{r_{i}^{2}}{n})}{1-\frac{1}{n}}$$

where *r*_*i*_ is the firing rate to the *i*^*th*^ pitch-shifted version of the syllable.

#### Syllable dissimilarity

For each pair of syllables used to measure the neuron’s SmI, we computed two distance metrics: (i) spectrogram distance and (ii) spectral feature difference. While spectrogram distance captures both temporal and spectral information of the stimulus, spectral features were computed specifically within a 55-ms window centered on the firing response (the peak response to the combined stimulus, averaged over repetitions) to examine how stimuli near the spike influenced summation.

The spectrogram distance was the Euclidean distance between flattened spectrograms of syllables. Spectrograms were generated using the stft function in Librosa, with *nfft* = 1024 and a Hanning window with 75% overlap. The second metric, the spectral feature difference, was the Euclidean distance between feature vectors consisting of spectral mean, quartiles, and standard deviation of the sound waveform. These spectral characteristics were calculated using the BioSound class from the soundsig package.

To calculate the temporal correlation between syllables, we computed the Pearson correlation coefficient between their respective power profiles within the same 55-ms window. These profiles provide a temporal representation of the overall energy of the syllable at each time point, and were obtained by averaging the spectrogram across frequency bins for each sound signal.

#### Regressing SmI on response and feature properties

A mixed-effects linear regression was fit to account for the contribution of individual factors to SmI. For syllable pair *i* presented to neuron *j*, this was described as:

(3) $$SmI_{ij}=\alpha_{j}+\beta_{1}\cdot {\left(R_{a}+R_{b}\right)}_{ij}+\beta_{2}\cdot adapt_{ij}+\beta_{3}\cdot \;distance_{i}+\beta_{4}\cdot \;temporal_{i}+\epsilon_{ij}$$

where the fixed effects, each associated with a coefficient *β*, are the predictors in the model: (i) sum of responses to individual syllables, (*R*_*a*_ + *R*_*b*)*ij*_, (ii) degree of cross-stimulus adaptation *adapt*_*ij*_, (iii) spectral feature distance *distance*_*i*_ and (iv) syllable temporal correlation *temporal*_*i*_. The residual errors *ϵ*_*ij*_ are assumed to follow a normal distribution: *ϵ*_*ij*_ ~ 𝒩 (0, *σ*^2^). The varying intercept *α*_*j*_ accounts for neuron-specific (random) effects and is assumed to be drawn from a distribution: $\alpha_{j}\sim N(\mu_{\alpha },\sigma_{\alpha }^{2})$, where *μ*_*α*_ and $\sigma_{\alpha }^{2}$ are the mean and variance of the random effects, estimated from the data. SmI values were converted to a normal distributions by a Box-Cox transformation (exponent *λ* = 0.38). The model was fit using the lmerTest function in R, and p-values for predictor coefficients were calculated using Satterthwaite’s approximation.

Marginal *R*^2^ values quantifying the proportion of variance explained by the predictors were calculated using the partialR2 function in R for mixed-effects models.

All statistical analyses were performed in R.

### 2.6 Neuron model

We simulated the integration of complex USV stimuli with a simple neuron model connected to 10 cylindrical dendritic subunits. The sound stimulus was first converted into spike trains which were inputs to the dendritic tree of a central auditory neuron. Code for the model is available at https://gitlab.com/kozlovlabcode/flex_integration.git.

#### 2.6.1 Stimulus encoding

To encode the sound stimulus into spikes, we used a neuron model based on methods described in [20] for simulating cochlear filtering. Sounds were passed through a bank of 3000 gammatone filters evenly spaced on the Equivalent Rectangular Bandwidth (ERB) scale. The center frequencies of these filters ranged between 30 - 120 kHz, which encompassed the frequency content of USV syllables used in the experiments. The filtered sounds, *x* (sound pressure in pascals), were subjected to half-wave rectification and compression to generate a current $I_{stim\;}=3{\left({\left[x\right]}^{+}\right)}^{\frac{1}{3}}$. This current, together with noise $I_{noise\;}^{c}$, was fed as an input $I_{in}^{k}$ to a point neuron. The evolution of the membrane potential, $V_{m}^{k}$, where the superscript *k* refers to either the stimulus-encoding neuron (denoted as *c*) or a neuronal compartment (as detailed in the following section), follows the dynamics of a leaky integrate-and-fire model with the standard form:

(4) $$C_{m}^{k}\frac{dV_{m}^{k}}{dt}=-g_{L}^{k}(V_{m}^{k}-E_{L}^{k})+I_{in}^{k}$$

where $C_{m}^{k},g_{L}^{k}$ and $E_{L}^{k}$ are the membrane capacitance, leak conductance and leak reversal potential, respectively.

When the membrane potential exceeds the threshold $V_{th}^{c}$, a spike is emitted and $V_{m}^{c}$ is reset to the reset potential, $V_{r}^{c}$, where it is held for a refractory period of 5 ms.

The noise current in the stimulus-encoding neuron $I_{noise\;}^{c}$ with standard deviation $\sigma_{noise\;}^{c}$ was implemented as:

(5) $$I_{noise\;}^{c}=\sigma_{noise\;}^{c}\sqrt{2\tau_{m}}\xi$$

where *ξ* is Gaussian noise with zero mean and unit s.d., and *τ*_*m*_ is the membrane time constant.

A total of 3000 such neurons made synaptic connections onto the dendrites of a central auditory neuron. Parameters for the stimulus encoding model are given in Table 2. This model was written using routines from the Brian2hears package [21].

#### 2.6.2 Compartmental central auditory neuron

The central auditory neuron in our model consists of a soma electrically connected to 10 uniform dendritic subunits. We implemented the model using the Dendrify framework and routines [22] for creating compartmental neuronal models with dendritic properties. Each compartment was modelled using an integrate-and-fire model as in Eq. 4. The inputs was $I_{in}^{s}$ to the soma *s* include an adaptive current *w*, while inputs $I_{in}^{d}$ coupling: to the dendrite *d* are driven by synaptic inputs and dendritic coupling:

(6) $$I_{in\;}^{s}=I_{axial\;}^{s}+I_{noise\;}^{s}-w$$

(7) $$I_{in}^{d}=I_{axial\;}^{d}+I_{syn}^{d}$$

The axial current, $I_{axial\;}^{k}$, is the current that flows between connected compartments. It is a function of the voltage difference between a compartment and its neighbour, multiplied by the coupling conductance $g_{c}^{k}$. In the somatic compartment this is the sum of the currents flowing into and from all the dendritic branches denoted by *D*:

(8) $$I_{axial\;}^{s}=\sum_{d\in D}g_{c}(V_{m}^{d}-V_{m}^{s})$$

and in the dendrite, $I_{axial\;}^{d}$ is:

(9) $$I_{axial\;}^{d}=g_{c}(V_{m}^{s}-V_{m}^{d}).$$

where $V_{m}^{d}$ and $V_{m}^{s}$ denote the dendritic and the somatic membrane potential, respectively.

The adaptive current *w* to the soma captures the transient spiking behaviour of auditory neurons recorded in the experiments. The spiking mechanism is different from that of the stimulus-encoding neuron in that when the membrane potential exceeds the threshold, $V_{th}^{s}$, it is stepped to *V*_*spike*_ for the neuron to spike. Each time the neuron spikes, *w* is increased by a constant current, *b*, that accounts for spike-triggered adaptation. After a short delay, the membrane potential resets to $V_{r}^{s}$. The spiking and reset events are described as follows:

(10) $$\;if\;V_{m}^{s}>V_{th\;}^{s}\;then\;\{\begin{array}{l} V_{m}^{s}\to V_{spike\;}\\ w\to w+b \end{array}$$

$$\text{if}\;t=t_{spike\;}+0.2ms\;then\;V_{m}^{s}\to V_{r}^{s}\text{.}\;$$

The dynamics of *w* follow:

(11) $$\tau_{w}\frac{dw}{dt}=a(V_{m}^{s}-E_{L}^{s})-w$$

where *a* is a coupling parameter determining how sensitive the adaptation current is to the membrane potential.

The current $I_{noise\;}^{s}$ to the soma is coloured noise with standard deviation $\sigma_{noise\;}^{s}$, and time constant *τ*_*noise*_ :

(12) $$\tau_{noise\;}\frac{dI_{noise\;}^{s}}{dt}=-I_{noise\;}^{s}+\sigma_{noise\;}^{s}\sqrt{2\tau_{noise\;}}\xi$$

For the dendritic compartment, $I_{syn}^{d}$ is the sum of synaptic currents entering through AMPA and NMDA receptors, where the subscript *syn* denotes the type of conductance (either AMPA or NMDA).

(13) $$I_{syn}^{d}=g_{syn}\cdot s_{syn}^{d}(V_{m}^{d}-E_{syn})\cdot \sigma (V_{m}^{d})$$

*g*_*syn*_ is the conductance for either AMPA or NMDA, *E*_*syn*_ the reversal potential of the receptor current, and *σ*(*V*) describes the voltage-dependent magnesium block of the NMDA receptor:

(14) $$\sigma (V_{m}^{d})=\frac{1}{1+\frac{\left[Mg^{2+}\right]}{\beta }\cdot exp(-\alpha (V_{m}^{d}-\gamma ))}$$

with magnesium concentration [*Mg* ^2+^] = 1*mM*, *β* = 3.57*mM*, *α* = 0.062*mV* ^−1^ and *γ* = 0*mV*.

The time course of the synaptic conductance is governed by two equations describing the rise and decay phase with independent time constants $\tau_{syn\;}^{rise\;}$ and $\tau_{syn}^{decay\;}$:

(15) $$\frac{ds_{syn}^{d}}{dt}=-\frac{s_{syn}}{\tau_{syn}^{decay}}+x$$

(16) $$\frac{dx}{dt}=-\frac{x}{\tau_{syn}^{rise}}+w_{syn}\cdot \delta (t-t_{f})$$

where *w*_*syn*_ is the synaptic weight and *δ*(*x*) is the Dirac delta function, and *t*_*f*_ is time of arrival of the presynaptic spike.

##### Compartment-specific properties

For a compartment with surface area *A*^*k*^, specific capacitance *c*_*m*_ and specific membrane resistivity *r*_*m*_, the membrane capacitance $C_{m}^{k}$ and leak conductance $g_{L}^{k}$ are:

(17) $$C_{m}^{k}=c_{m}A^{k}$$

(18) $$g_{L}^{k}=\frac{1}{r_{m}}A^{k}$$

All parameters for the auditory neuron model are shown in Table 3.

#### 2.6.3 Quantifying spatial and temporal overlap on dendritic branches

We constructed spatial and temporal profiles of synaptic inputs to the dendritic branches by summing the AMPA synaptic conductance, *s*_*AMPA*_, across branches and time respectively. To quantify the degree of input overlap across branches and across time, we computed two Pearson correlation coefficients for each syllable, one between the spatial profiles and another between the temporal profiles. As shown in Table 4, the median correlation value was used as a threshold to categorize inputs as dispersed and (temporally) correlated, and as clustered and uncorrelated.

## 3 Results

### 3.1 MAX and AND operations in mouse auditory neurons

**Figure 1:**
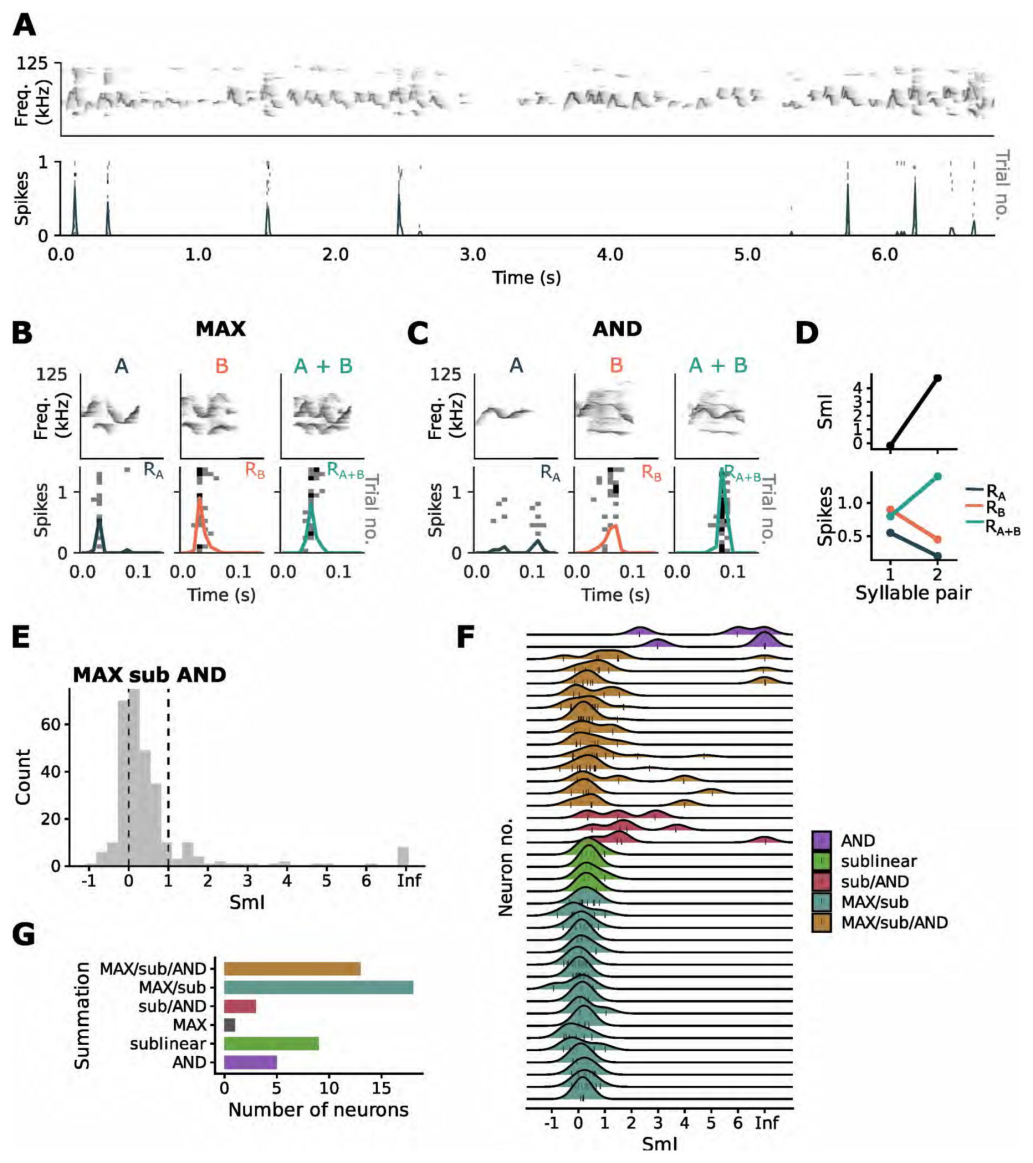
Probing feature recombination functions in single auditory neurons. (A) USV-responsive units were first identified for the experiment by presenting USV songs for several repetitions. Example PSTH (solid trace) from a single unit responding to specific USV syllables. The PSTH is overlaid over a raster plot showing spike responses over trials. (B) Pairs of syllables evoking responses in the unit were extracted and presented to the unit either separately or superimposed. Showed here are responses of the same unit in (A) to individual syllables (left and middle panels) and to their superimposition (right panel). The unit responds in a MAX-like manner to this combination of syllables. (C) Same unit showing an AND-like response to a different combination of USV syllables. (D) Firing rates to the syllables in (B) and in (C) and the resultant SmI. (E) Distribution of SmIs across all units. (F) SmIs for individual units, measured in response to different stimulus pairs. Labels correspond to the types of recombination functions exhibited by each unit. Shown here are units for which multiple SmIs were recorded. (G) A total of 49 units were recorded from auditory cortex of 21 mice. Most units exhibited more than one type of recombination function

To probe the computations of single neurons in mouse auditory cortex, we recorded spiking responses to mouse ultrasonic vocalizations (USVs). We first identified units that were responsive to USVs and extracted syllables (~ 100 ms in duration) coinciding with peaks in the Peristimulus Time Histogram (PSTH) (Figure 1A). Syllables extracted from the continuous USV song were presented either individually or superimposed with an appropriate time lag. The time lag was chosen to evoke the maximal response when two syllables were combined, so as not to miss the optimal temporal window for integration. For some syllable sets, the response to the combination of the two syllables was equal to or less than the response to either syllable, indicating a MAX-like response (Figure 1B). We also observed AND-like responses, where sub-threshold individual syllables would combine to evoke a response (Figure 1C), or when the superimposed syllables produced a firing rate that was greater than the arithmetic sum of the individual responses. These responses were quantified using the summation index (SmI; Eq 1). SmIs close to 0 correspond to the MAX-like operation and values equal to and greater than 1 indicate AND-like summation of inputs.

**Figure 2:**
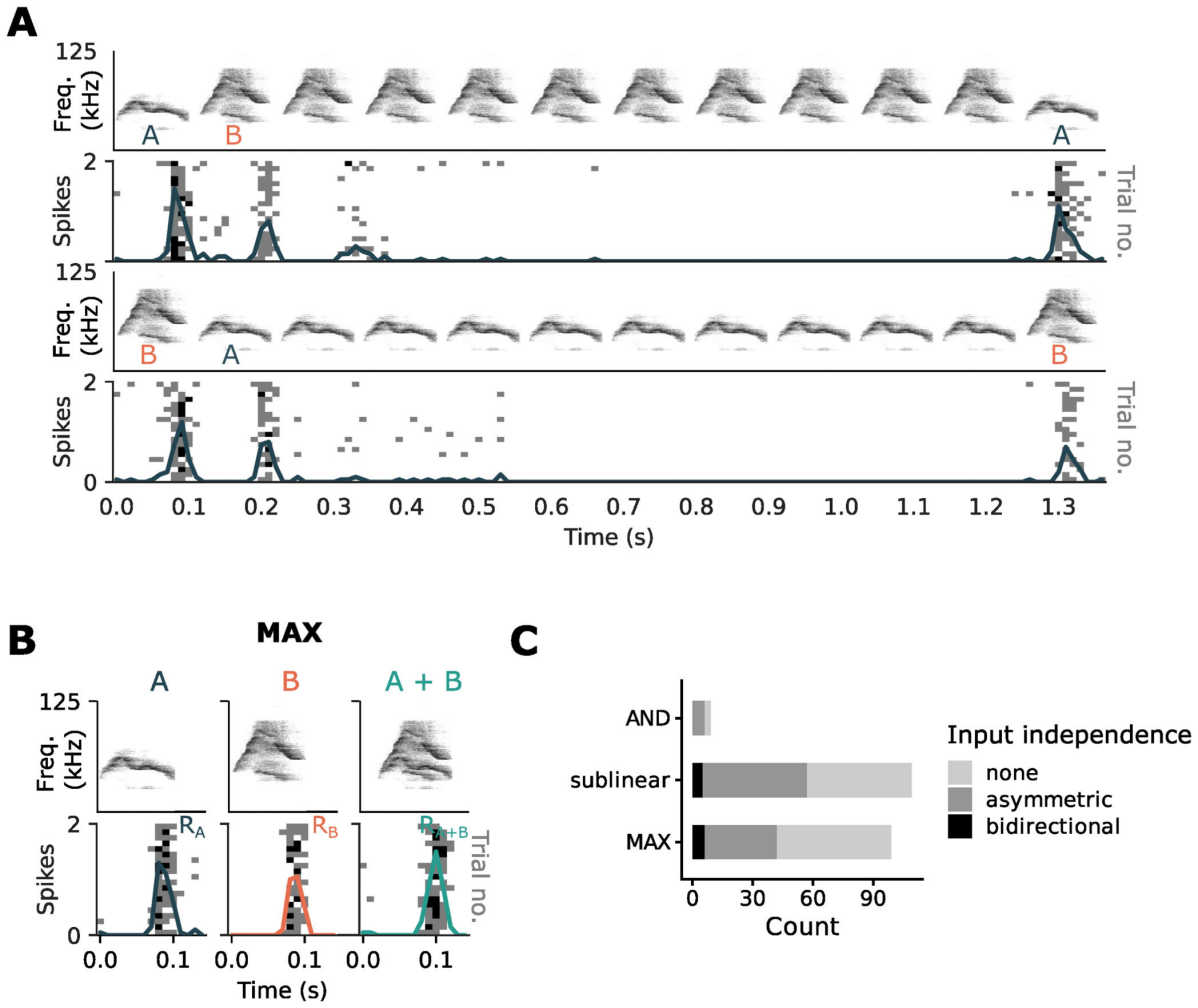
Testing input independence of pairs of syllables. (A) Cross-adaptation protocol. Example unit showing asymmetric input independence where there was a robust response to the first syllable before and after repeated presentations of the second, but not when the order of the two syllables was reversed. (B) Combination of the two syllables in (A) produced a MAX-like response by the unit. (C) Breakdown of input independence for different recombination functions.

**Figure 3:**
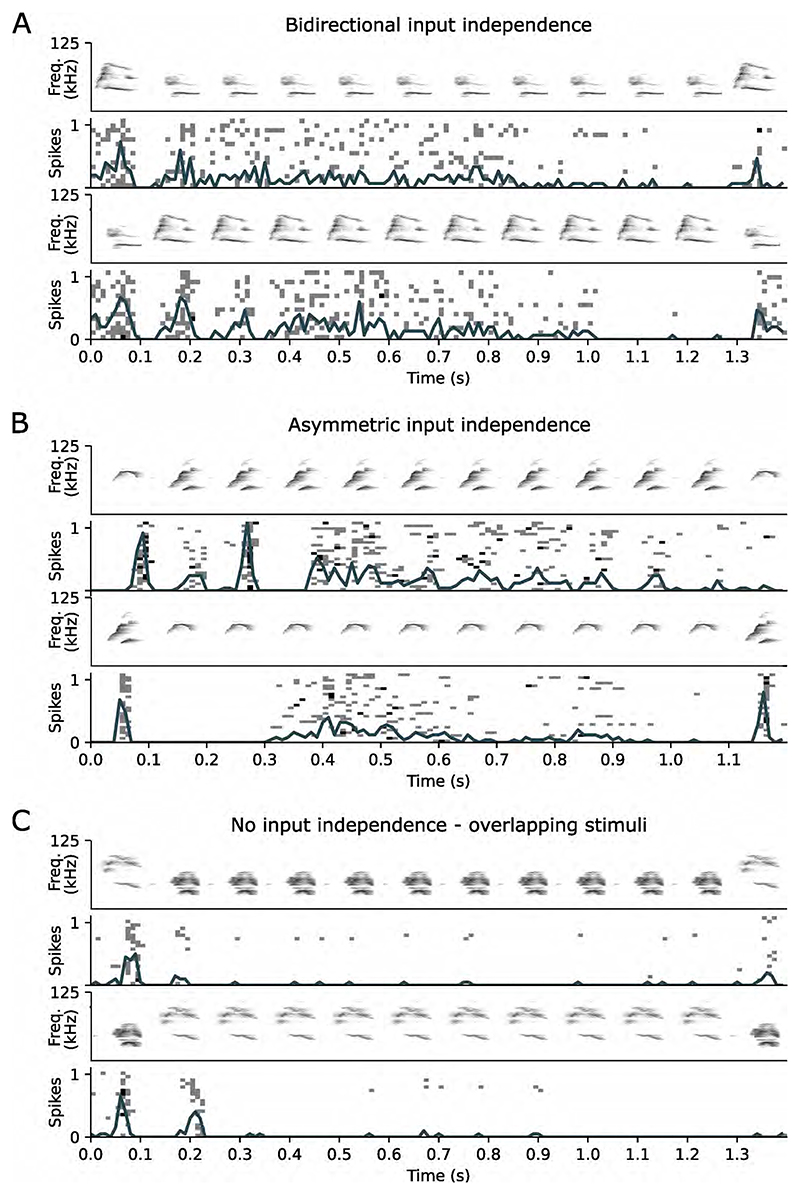
Examples of input independence and cross-adaptation. (A) Bidirectional input independence: preservation of ≥ 60% of the original response after adaptation to repeated presentations of the other syllable. (B) Asymmetric input independence: preserved response to the deviant syllable in one sequence but not in the reverse order. (C) Cross-adaptation: presentation of the deviant syllable fails to elicit a response after adaptation to the other syllable.

A set of syllables activating the neuron through shared inputs would also produce a reduced response that resembles the MAX-like operation, biasing SmI values toward 0 due to input saturation. To distinguish true MAX-like operations from such apparent MAX effects arising from overlapping inputs, we performed an additional control testing whether each set of syllables activated the neuron through independent inputs [16]. Under a protocol to test cross-adaptation (Section 2.4), we measured the recovered response to one syllable after repeated presentations of the other syllable in the pair to cause adaptation. The order of the two syllables was then reversed in the following sequence (Figure 2A). We observed different degrees of input independence across recombination functions (Figure 2C), with pairs of stimuli exhibiting input independence either in both sequences (bidirectional input independence), in only one sequence (asymmetric), or not at all (no input independence; Figure 3). In 99 MAX responses recorded, 42 exhibited some degree of input independence where adaptation to repetitions of one syllable preserved at least 60% of the response to the other (deviant) syllable, either in one sequence (asymmetric input independence; *n* = 36) or in both sequences (bidirectional input independence; *n* = 6).

### 3.2 Single-neuron flexibility in input integration

**Figure 4:**
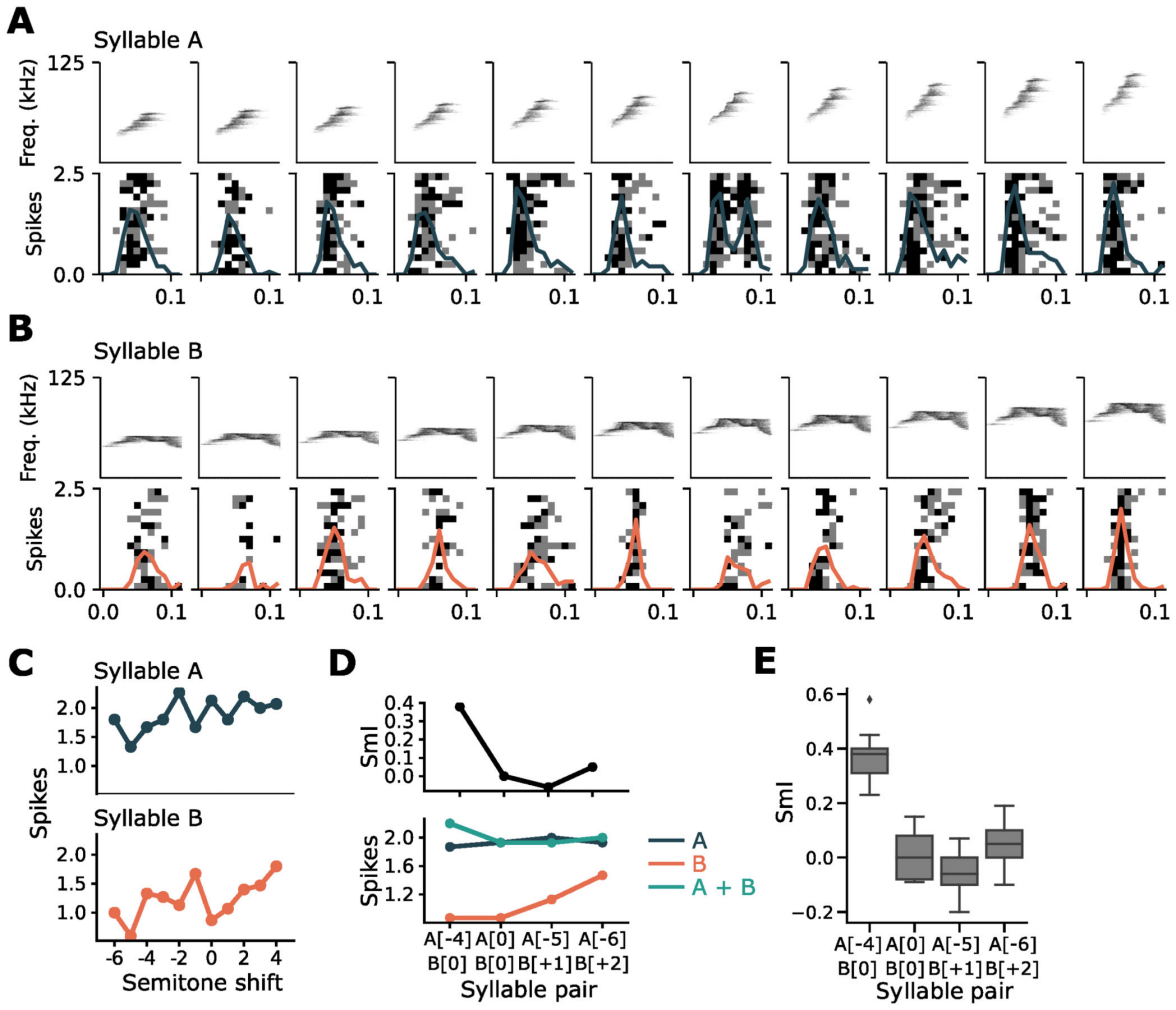
Integration of pitch-shifted inputs. (A) and (B) We tested the recombination function of a unit to different variations of the same syllable combination. Shown here are responses to pitch-shifted versions of two syllables. This unit retains good responsiveness to changes in pitch. (C) Tuning curves to pitch-shifted versions of the two syllables. (D) (Top) SmIs for pitch-shifted versions of the syllables shown in (A) and (B). (Bottom) Corresponding firing rates to individual and combined syllables; pitch-shift indicated in square brackets. (E) Variability in SmIs for each syllable pair calculated using a leave-one-trial-out method.

Across all units, we observed a range of summation behaviors, with the peak of the distribution of summation indices close to 0 (Figure 1E), suggesting a predominance of the MAX-operation in this population of cells. Individual units exhibited a range of summation behaviours for different syllable pairs (Figure 1F) and were capable of implementing more than one type of recombination function (Figure 1G). To investigate changes in the neuron’s integration behaviour to simple feature transformations, we pitch-shifted (up and down by 6 semitones) syllables of each pair (Figure 4A). Some units were broadly tuned and retained responses to the pitch-shifted syllables (Supplementary Figure S1A), while other units responded selectively to syllables pitch-shifted within a limited range (Supplementary Figure S1B). Combining pitch-shifted versions of the same base pair of syllables altered the neuronal SmI (Figure 4D). Hence a neuron’s summation behavior, as measured by the SmI, changes in an input-dependent manner, highlighting the flexibility of single neurons in implementing different integration operations. To explore whether SmIs vary from trial to trial for the same stimuli, we calculated the variability in SmI for an example neuron using a leave-one-trial-out method. The standard deviation in SmIs across trials for each stimulus pair was around 0.1, suggesting that recombination functions can be flexible even for the same stimuli across different trials due to noise (Figure 4E). Such variability may influence the probability that a neuron implements a particular recombination function on a given trial.

### 3.3 Factors influencing stimuli integration

As the integration of USV syllables spanned the sublinear to supralinear regimes, we wondered what factors determined the SmI. Through our analyses, we found that the activation strength of inputs, and the degree of dissimilarity between inputs influence the neuron’s integration response.The arithmetic sum of responses to individual syllables (*R*_*a*_ + *R*_*b*_) correlated negatively with SmI values (Supplementary Figure S2A; Spearman’s *ρ*_(*n*=280)_ = −0.42, *p* < 0.001). This suggests that integration was more MAX-like with strongly activating inputs and more AND-like with weakly activating or subthreshold inputs. Degree of cross-stimulus adaptation (the neuron’s response to a syllable after adapting to the other syllable in the pair) correlated weakly with SmI (Supplementary Figure S2B; Spearman’s *ρ*_(*n*=280)_ = 0.18, *p* = 0.003), with more independent inputs eliciting a higher, more AND-like, SmI.

To examine if tuning properties of the neuron affected SmI, we characterized the selectivity of neuronal responses to pitch-shifting using a sparsity index (Eq 2). For each pair of syllables contributing to the SmI, two sparsity indices were calculated, each representing the degree of selectivity to pitch-shifted versions of the respective syllable (Supplementary Figure S1C). A positive correlation was observed between SmI and pitch selectivity, which was more pronounced for the smaller (less sparse) index in each pair (Supplementary Figure S1D; *ρ*_(*n*=183)_ = 0.35, *p* < 0.001) compared to the higher (more sparse) index (*ρ*_(*n*=183)_ = 0.25, *p* < 0.001). Integration tends to be more AND-like when the neuron exhibits greater pitch selectivity for a given syllable.

Besides response properties, we examined if acoustical properties of the stimuli could affect a neuron’s SmI. Specifically, we were interested in whether feature integration was biased by how acoustically distinct the stimuli were, which would have implications for object recognition computations. We characterized syllable dissimilarity using two metrics, calculating the distance between either (i) flattened spectrograms or (ii) feature vectors consisting of syllable spectral properties (frequency quartiles, mean frequency and bandwidth).

Comparing MAX-like, sublinear and AND-like SmIs, there was an overall difference in the degree of syllable dissimilarity as measured by spectrogram distance (Supplementary Figure S2C; Aligned ranks transformation ANOVA with observations grouped by neuron, *F*_(2,283.6)_ = 4.16, *p* = 0.017). Post-hoc analyses with Tukey’s pairwise-comparison test showed that this difference was between MAX-like and sublinear SmIs (*t*_(250)_ = −2.796, *p* = 0.015), indicating that the MAX-like operation may be employed when inputs are more similar in spectrotemporal content. Although the distance between spectrogram pairs producing AND-like SmIs appeared to be greater than that of MAX-like SmIs, this difference was not statistically significant (*t*_(277)_ = −1.069, *p* = 0.53).

A traditional approach for comparing and characterizing vocalizations is to extract certain acoustic features from the stimulus waveform [23]. Syllables were represented using feature vectors and the distance between vectors of a pair was used as the second metric of syllable dissimilarity. As sensory coding models [24] assume that spiking is determined by recent stimuli, spectral features were extracted from a 55-ms window of the firing response (the peak of the PSTH). We found a weak positive correlation between spectral feature distance and SmI (Supplementary Figure S1D; *ρ*_(*n*=280)_ = 0.22, *p* < 0.001). This relationship was more evident for pairs of stimuli that were temporally anti-correlated (i.e., correlation between power profiles over time < 0, see methods); *ρ*_(*n*=91)_ = 0.4, *p* < 0.001). These results indicate that the integration of dissimilar features tends towards more AND-like summation.

To assess the relative importance of these various factors influencing the SmI, we fit a mixed-effects linear regression model with response and feature properties as regressors. The coefficients of the regressors for the sum of individual responses (*R*_*a*_ + *R*_*b*_ ;*t*_(114)_ = −7.107, *p* < 0.001), spectral feature difference (*t*_(274)_ = 4.78, *p* < 0.001), and temporal correlation (*t*_(270)_ = −2.43, *p* = 0.016) were found to be significant. The degree of cross-adaptation had no significant impact on the SmI (*t*_(269)_ = 1.44, *p* = 0.15). The sum of individual responses accounted for the largest proportion of variance in the model (20%), spectral feature distance and feature temporal correlation accounted for a further 12%. These results are shown in Supplementary Tables S1 and S2.

### 3.4 Investigating single-neuron flexibility in a biophysical neuron model

**Figure 5:**
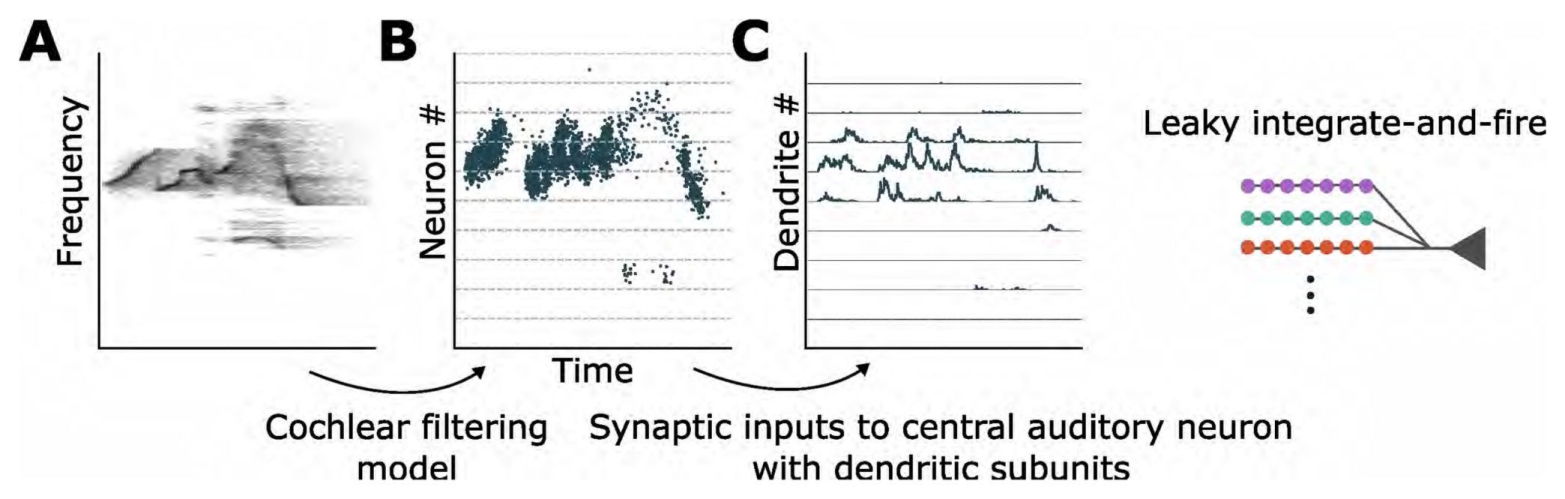
Encoding sound stimuli into spike trains. (A) Example spectrogram of a USV clip. (B) Spike train representation of the sound stimulus after processing with the auditory periphery model. Neurons with a smaller index encode low frequencies. (C) Synaptic inputs arriving at each dendrite. Each dendrite is connected to 300 neurons from (B) in the corresponding frequency band.

As our experimental results showed that neuronal response properties and feature characteristics can influence the SmI, we turned to a simple model of dendritic integration to explore the mechanisms underpinning these dependencies. To approximate sound encoding in the auditory periphery, sound clips were filtered through a bank of 3000 gammatone filters with center frequencies spanning 30 kHz - 120 kHz on the ERB (Equivalent Rectangular Bandwidth) scale. The filtered sound from each channel was fed as an input current into a noisy leaky integrate-and-fire neuron, producing a spike train representation of the stimulus for a certain frequency band. Neurons encoding the stimuli were connected to an adaptive integrate-and-fire ’central auditory’ neuron with ten dendrites. Each dendrite received synaptic contacts from 300 encoding neurons such that similar frequencies converged on the same dendrite (Figure 5). All syllable combinations used in the experiments, superimposed with a range of time overlaps (from 0 - 20 ms), were presented to the model. This produced a distribution of SmIs (*n* = 730) with a peak at 0 corresponding to the MAX operation (Figure 7A). Selecting the optimal time overlap which produced the highest integration response for each syllable combination (*n* = 146), as was done in the experiments, revealed more AND-like responses. This shows that the temporal alignment of stimuli affects the SmI distribution and is an important step in probing neuronal integration.

**Figure 6:**
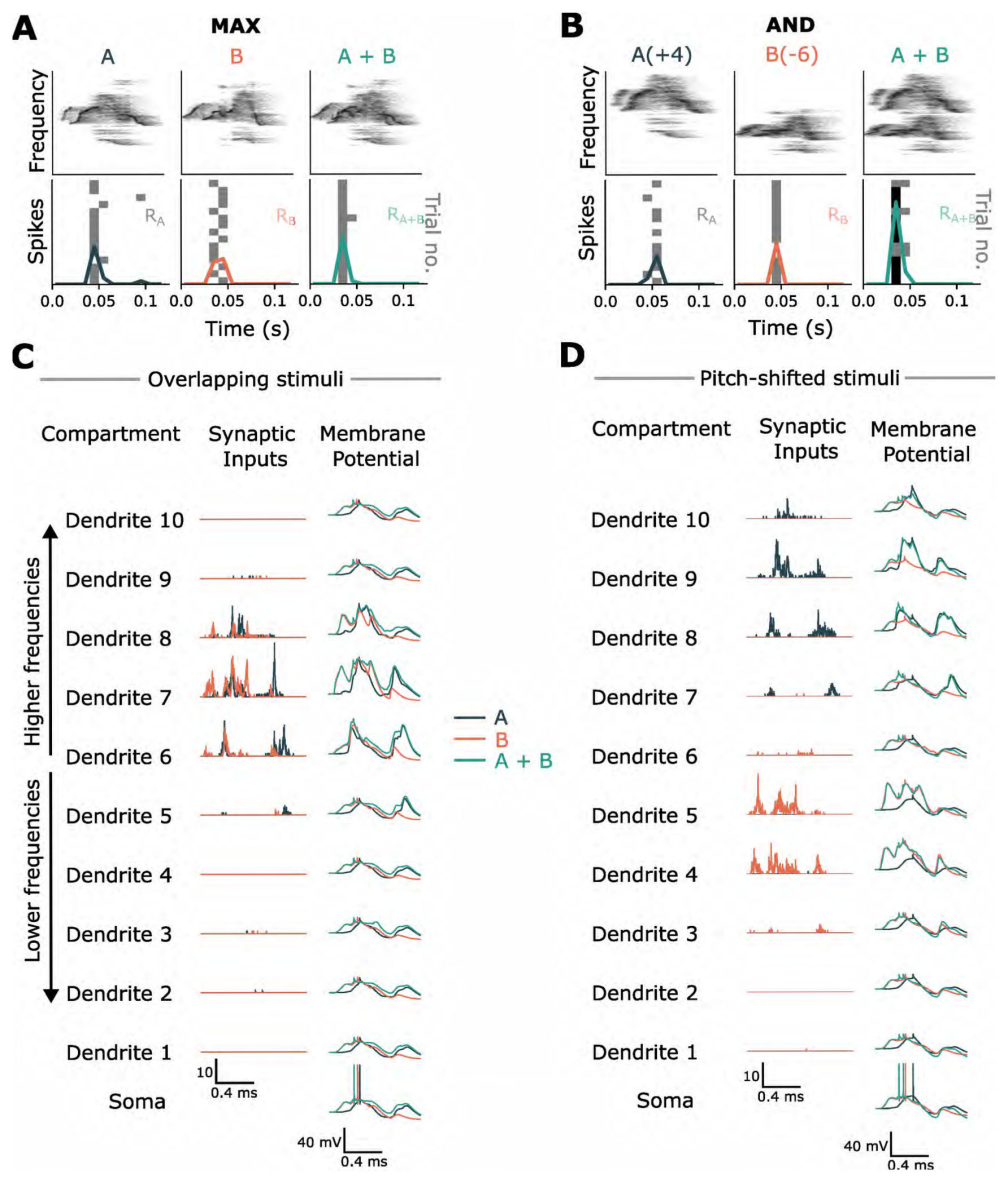
The spatiotemporal pattern of inputs on passive dendrites affects the resultant feature recombination function. (A) The MAX-like operation observed in the model for similar inputs. Top panels: Spectrograms of two syllables presented to the model. Lower panels: Spiking response of the model to two syllables, presented either in isolation or combined, over 15 trials. A small noise current injected to the soma of the neuron produces trial-to-trial variability. (B) Similar to (A) but with clip ’A’ shifted up by 4 semitones and clip ’B’ shifted down by 6 semitones to increase their spectral separation. This produces and AND-like response in the model. (C) Inputs that overlap in spectral content converge on the same dendrite, producing a sublinear response. Shown here are the dynamics in each compartment for the set of stimuli shown in (A). Each dendrite of the central auditory neuron receives inputs from 300 coclear filterbank neurons, with each dendrite encoding a distinct frequency range within the 30 - 120 kHz spectrum of the sound stimulus. Center column: Spatiotemporal profile of synaptic activations on each dendrite for the individual syllables. Right column: Membrane potential recorded in the dendritic and somatic compartments. The neuron emits a single spike (somatic compartment, green trace) when integrating temporally coincident and spectrally overlapping inputs. (D) Inputs separated in spectral content target different branches and are integrated linearly, leading to an enhanced response in comparison (neuron emits two spikes).

**Figure 7:**
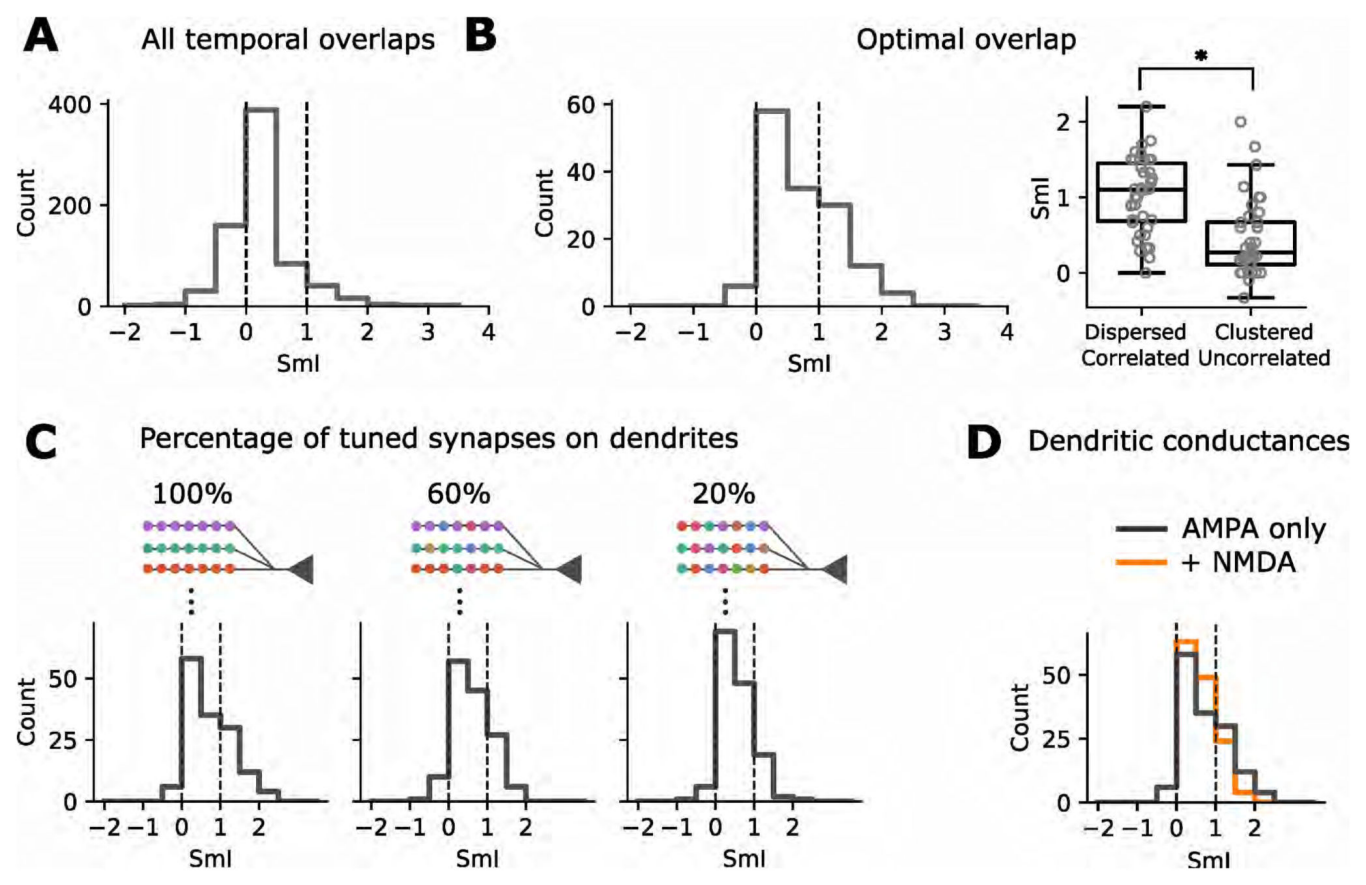
The SmI distribution changes with dendritic conductances and input mixing. (A) Distribution of SmIs for all syllable combinations, combined with different time overlaps and (B) only with the optimal time overlap, as in the experiments. (right) Boxplots of SmIs, for dispersed and temporally correlated inputs, versus clustered and uncorrelated inputs. (C) Distribution of SmIs when the degree of frequency tuning homogeneity on dendrites was varied in the passive model. We began with a structured synaptic arrangement (left), where inputs to each dendrite encode a specific frequency range of the stimulus. We then transitioned to more mixed patterns, by introducing inputs that were sampled from frequency bins outside the dendrite’s specific range. (D) Effect of adding NMDA receptors to the model on the SmI distribution.

In theory, the simultaneous activation of inputs spaced close together on passive dendrites yields a sublinear response, owing to the reduction in synaptic driving force from each depolarization and mutual shunting of inputs. Spatially separated inputs integrate linearly, resulting in a more enhanced response [25]. We wondered if these summation rules derived with simple synaptic inputs result in different feature recombination functions for similar versus dissimilar stimuli. As an example, we show that two syllables with input patterns that colocalize in time and space (branch) are integrated to produce a MAX-like response (Figure 6). Increasing the spectral distance between them (by pitch-shifting) produces an AND-like response. Inputs to the model that were spatially dispersed across branches and correlated in time had higher SmI values, compared to clustered and asynchronous inputs (Kruskal-Wallis test *χ*^2^ = 22.1, p < 0.001;, Figure 7B).

Our simulations were performed under structured connectivity, where all synapses on a given dendrite were co-tuned to a specific frequency range. Randomizing synapse locations across branches, to increase the heterogeneity of frequency tuning within individual branches, resulted in the distribution of SmIs becoming more MAX-like. The median SmI decreased from 0.635 to 0.425 and the interquartile range from 0.8 to 0.65 (Figure 7C). Thus far, all simulations were performed using a model of passive input integration. To explore how active dendritic properties influence the integration of inputs, we added NMDA conductance, which shifted the distribution toward more MAX-like SmIs (Figure 7D).

## 4 Discussion

One can explore how neurons integrate their inputs at different levels of abstraction. At the level of biophysical mechanisms, the interplay between passive cable properties and active dendritic conductances enables nonlinear and complex transformations over synaptic inputs. On a computational level, studies have demonstrated that neurons implement the biological equivalent of logical operations integral for pattern recognition. Our work attempts to bridge these two levels of explanation. We probed the neurons’ feature recombination functions experimentally with natural and ethologically relevant stimuli to explore how it combines real-world features. We used a computational model to explore how spatiotemporal patterns of synaptic inputs on passive dendritic branches influence the resultant integration operation of the neuron.

Together with our previous study [16], this work highlights the flexibility in single-neuron integration operations and heterogeneity across neurons. Such flexibility can arise at different stages of the mapping between stimuli and neural activity [26]. Neurons exhibit stimulus-dependent tuning and have distinct receptive fields for processing different stimulus classes [27–29]. Furthermore, the nonlinearity relating receptive field-filtered inputs to firing has also been shown to be dynamic, adapting to changes in environmental statistics [4, 30] and implementing normalization [31]. Here, we have focused on the input-output mapping under steady-state conditions, for instantaneous feature extraction.

Experimentally, we recorded an SmI distribution with a peak at zero, corresponding to the MAX-like operation. Our simple dendritic integration model, with each dendrite receiving frequency-tuned synaptic inputs and integrating inputs passively, produced a similar distribution, with most recombination responses tending towards sublinear and MAX-like responses. When examining the impact of stimulus characteristics on SmI responses, we found experimentally that the integration of strongly activating features tended to be more MAX-like, whereas greater dissimilarity between stimulus pairs biased the SmI towards more AND-like values. Consistent with this finding, in our computational model, pairs of stimuli that activated temporally coincident inputs distributed across different dendritic branches, produced integration responses that were more AND-like. This aligns with findings that dispersed synaptic activation across the dendritic tree produces larger somatic depolarizations compared to clustered synaptic activation [32], providing a mechanism by which spectrally distinct stimuli can evoke AND-like integration under passive dendritic conditions. In terms of computational implications, these results align with the design of pattern recognition models. A neuron may achieve invariance to distractors by responding to a preferred stimulus which drives it strongly, thereby performing the MAX operation over its inputs. However, when features are sufficiently distinct to form a new composite, inputs may be pooled additively to achieve selectivity.

Although not studied in this work, receptive field properties are likely to influence the type of computation performed by the neuron. Currently, our biophysical model uniformly represents all stimuli across time and frequencies, neglecting tuning-specific effects on integration. In reality, the bandwidth and temporal profile of the receptive field can alter the window of integration of inputs to the neuron. [11] show that the same cells perform different computations in different places of their visual field. Specifically, OFF ganglion cells switch between quasilinear and nonlinear modes of integration depending on the location of a moving bar in the receptive field. In addition, the simultaneous presentation of a bar in the receptive field center suppressed the response to a distant bar. Investigating how a neuron performs different computations for different regions of its spectrotemporal receptive field, in the context of auditory processing, presents an interesting avenue for future research.

Many studies have examined how the location, timing of inputs and dendritic conductances shape neuronal output [9, 33–36]. For passive dendrites, spatially distributed inputs integrate linearly, resulting in an enhanced response compared to sublinear integration within dendritic branches. In contrast, clustering inputs on active dendrites engages voltage-gated conductances, producing supralinear integration [36–38]. While these studies provided much insight into the mechanisms of neuronal integration, they were performed with well-controlled synaptic inputs. Under *in vivo* conditions, where naturalistic input patterns are complex and correlated, response predictions based on integrating simple stimuli may not hold [39, 40] and the effect of local dendritic nonlinearities may be smaller than expected [41]. The relatively modest effect of increasing spectral (and hence spatial) separation distance between input stimuli on SmI suggests that there are other factors that cannot be accounted for by simple cable properties of the neuron.

In our model, synapses were highly organized into functional clusters, so that the dendrites exhibited branch-specific frequency tuning. Inputs from spectrally dissimilar stimuli were segregated over several dendrites, whereas similar features converged onto the same branches, permitting different rules of integration to operate over uncorrelated and correlated inputs. In reality, the tuning of synapses on single dendrites is likely to be more heterogeneous [42–44]. More work is needed to investigate feature integration under less structured connectivity, coupled with the effect of voltage-gated dendritic conductances. Our simulations provide some indication that having an expansive nonlinearity (through the addition of NMDA receptors) in dendrites could underlie MAX-like computations, by boosting responses to individual stimuli.

Our study on single-neuron feature integration has important implications for network function and computation. The choice of activation functions in artificial neural networks can impact learning performance [45, 46], yet these functions are typically static in most networks. Multiplexing computations in single neurons could unlock interesting network properties and behaviors. Adaptive input-output functions have been postulated to confer robustness to perturbations, and bring recurrent neural network dynamics to the edge of chaos, which is optimal for information propagation [6]. Other hypothesized roles for these adaptive functions include statistical whitening transformations [47] and enabling multitask learning [5].

In conclusion, our study has sought to investigate how neurons transform inputs to perform computations important for pattern recognition. The integration of complex spatiotemporal inputs on the dendritic tree is explored as a candidate mechanism for the single-neuron flexibility observed in the experiments. Future studies on dynamic single-neuron integration embedded in networks will provide further insights into the functional consequences of this flexibility.

## Supplementary Material

Supplementary Material

## Figures and Tables

**Figure:**
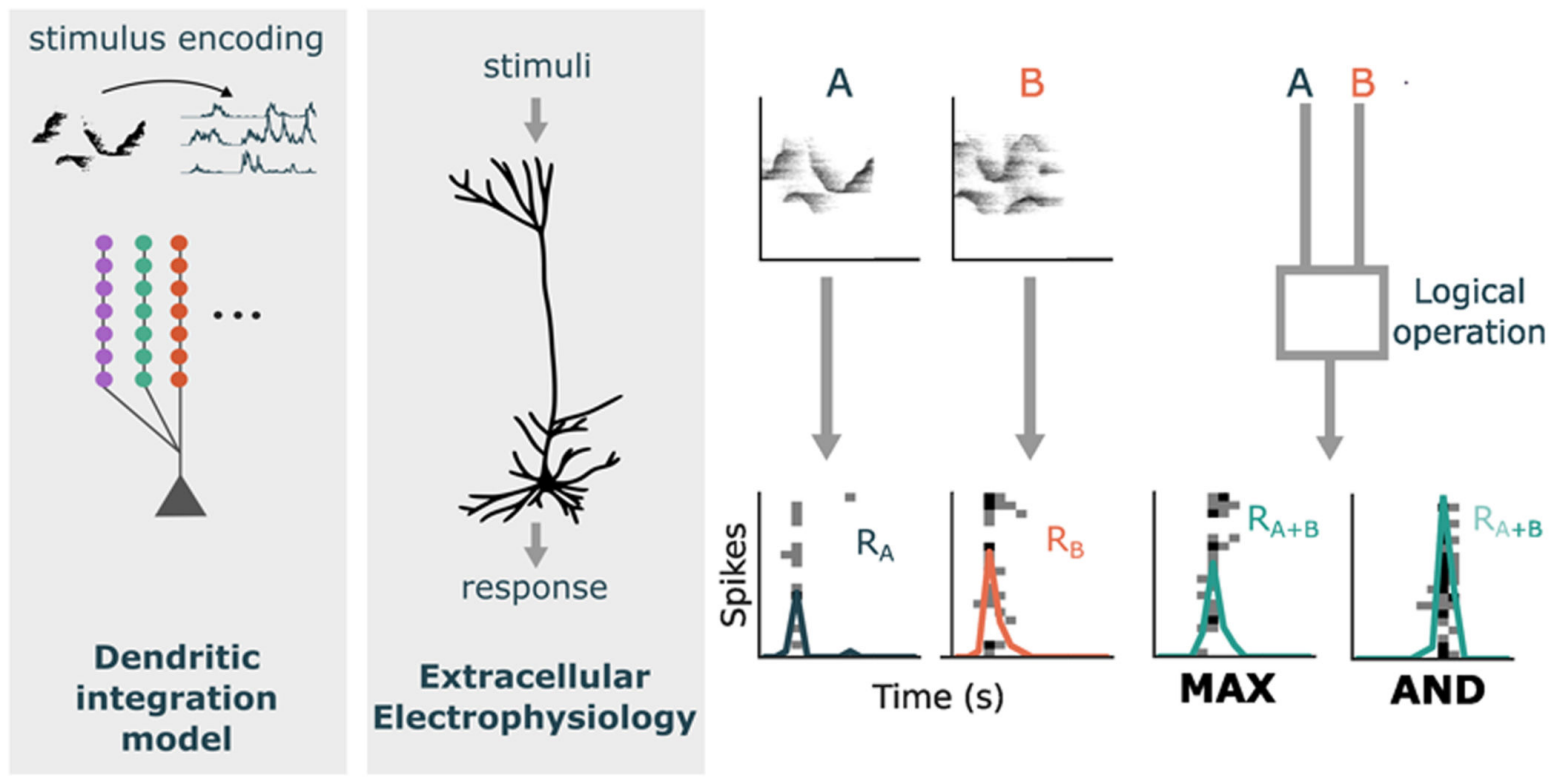


**Table 1:** The summation index and integration functions

SmI ≤ 0	MAX-like
0 < SmI < 1	Sublinear
SmI≈1	AND-like

**Table 2:** Encoding model parameters

Membrane capacitance	${\text{C}}_{m}^{c}$	1mF
Leak conductance	${\text{g}}_{L}^{c}$	1S
Membrane time constant	*τ_m_*	1ms
Resting potential	${\text{E}}_{L}^{c}$	- 60mV
Spiking Threshold	${\text{V}}_{th}^{c}$	- 40mV
Reset potential after spike	${\text{V}}_{r}^{c}$	- 20mV
Noise standard deviation	$\sigma_{noise\;}^{c}$	3mA

**Table 3:** Central auditory neuron parameters

Timestep	dt	0.1ms
Specific membrane capacitance	*C_m_*	*0.5 μ* *Fcm* ^−2^
Specific membrane resistance	*r_m_*	20 *kΩcm*^−2^
Axial resistance	r *_a_*	150 *Ωcm*
Resting membrane potential	E *_L_*	-60mV
Spiking threshold	${\text{V}}_{th}^{s}$	-50mV
1*^st^* voltage reset	V *_spike_*	0mV
2*^nd^* voltage reset	${\text{V}}_{r}^{s}$	-58mV
Soma length		25 *μm*
Soma diameter		25 *μm*
Dendrite length		100 *μm*
Dendrite diameter		1 *μm*
Area scale factor		3
Spine area factor		1.5
Noise time constant	*τ_noise_*	20ms
Noise standard deviation	*σ_noise_*	35pA
Coupling conductance	g *_c_*	0.8nS
Adaptation current	b	200pA
Adaptation coupling to membrane potential	a	0.8nS
Adaptation time constant	*τ* *_w_*	100ms
AMPA conductance	*g_AMPA_*	0.8nS
NMDA conductance	*g_NMDA_*	0.8nS
AMPA rise time	$\tau_{AMPA}^{\text{rise}\;}$	0.2ms
AMPA decay time	$\tau_{AMPA}^{decay\;}$	3ms
NMDA rise time	$\tau_{NMDA}^{rise\;}$	2ms
NMDA decay time	$\tau_{NMDA}^{decay\;}$	60ms
AMPA reversal potential	*E_AMPA_*	0mV
NMDA reversal potential	*E_NMDA_*	0mV
AMPA weight	*w_AMPA_*	0.5
NMDA weight	*w_NMDA_*	0.05

**Table 4:** Synaptic input patterns in the neuron model

Label	Temporal Correlation	Spatial Correlation
Dispersed and correlated	High (> 0.3)	Low (< 0.1)
Clustered and uncorrelated	Low (< 0.3)	High (> 0.1)

## New and Noteworthy

Sensory neurons compute over their inputs, combining stimuli to achieve selectivity and invariance for pattern recognition. Using real-world stimuli, we show that cortical neurons are flexible, capable of implementing more than one computation. We investigate this flexibility by modeling how synaptic activation patterns of real-world stimuli affect dendritic integration and resultant neuronal computation. Our work bridges the gap between biophysical mechanisms and computation, linking neuronal input integration to pattern recognition.
