# Supplementary material for: Flexible integration of natural stimuli by auditory cortical neurons
Source: J Neurophysiol. Author manuscript; Available in PMC 2026 May 21. (PMC7619095; doi:10.1152/jn.00200.2025)
Supplement: Supplementary Material [file EMS213586-supplement-Supplementary_Material.docx]

Supplemental material

**Supplemental Figures:** S1–S2

**Supplemental Tables:** S1–S2

All supplemental materials are available at:

https://figshare.com/articles/figure/Supplementary_figures/31043986
